# Mediterranean Personalized Diet Combined with Physical Activity Therapy for the Prevention of Cardiovascular Diseases in Italian Women

**DOI:** 10.3390/nu12113456

**Published:** 2020-11-11

**Authors:** Laura Di Renzo, Giulia Cinelli, Maria Dri, Paola Gualtieri, Alda Attinà, Claudia Leggeri, Giuseppe Cenname, Ernesto Esposito, Alberto Pujia, Gaetano Chiricolo, Chiara Salimei, Antonino De Lorenzo

**Affiliations:** 1 Section of Clinical Nutrition and Nutrigenomic, Department of Biomedicine and Prevention, University of Rome Tor Vergata, 00133 Rome, Italy; paola.gualtieri@uniroma2.it (P.G.); delorenzo@uniroma2.it (A.D.L.); 2 Department of Biomedicine and Prevention, University of Rome Tor Vergata, 00133 Rome, Italy; albpujia@gmail.com (A.P.); nucciochiricolo@gmail.com (G.C.); 3 School of Specialization in Food Sciences, University of Rome Tor Vergata, 00133 Rome, Italy; giuliacinelli88@gmail.com (G.C.); alda.attina@gmail.com (A.A.); claudialeggeri@gmail.com (C.L.); 4 Predictive and Preventive Medicine Research Unit, Bambino Gesù Children’s Hospital, IRCCS, 00165 Rome, Italy; 5 Department of Surgical Sciences, School of Applied Medical-Surgical Sciences, University of Rome Tor Vergata, 00133 Rome, Italy; marystenr@hotmail.it; 6 Comando Generale Arma Carabinieri, Direzione di Sanità, 00197 Rome, Italy; giuseppe.cenname@carabinieri.it; 7 Department of Human Policies (General Directorate) of Basilicata Region, 85100 Potenza, Italy; ernesto.esposit1962@gmail.com; 8 Department of Systems Medicine, University of Rome Tor Vergata, 00133 Rome, Italy; chiara.salimei@gmail.com

**Keywords:** cardiovascular disease, Mediterranean diet, non-communicable disease, obesity, physical activity

## Abstract

Cardiovascular diseases (CVDs) and inflammatory risk indexes are used to calculate the exposure to morbidity. Most of them are suggested by the American College of Cardiology/American Heart Association to predict the risk of CVDs diagnosis in primary prevention, instead of treating the ongoing pathology. Prevention starts from habit changes with the prescription of diet and physical activity (PA). The aim of the study is to investigate the effectiveness of a personalized Mediterranean Diet (MD) and a PA intervention, on the risk indexes Atherogenic Index of Plasma (AIP), Lipid Accumulation Product (LAP) and Fatty Liver Index (FLI) in a population of women at risk of CVDs with different pathological conditions. After treatment, patients achieved the best results in body composition (BC) and laboratory tests. The BC analysis showed a significant reduction of total body Fat Mass (FM). CVDs risk indexes significantly decreased, except for Neutrophil/Lymphocyte (NLR) and Platelet/Lymphocyte Ratios (PLR). The reduction of the CVDs indexes associated with lipid profile was linked to both weight and FM decrease. AIP and LAP were significantly reduced when losing fat mass and body weight, respectively. A personalized MD therapy plus a PA program led to body weight loss, BC remodelling and risk indexes reduction.

## 1. Introduction

The World Health Organization (WHO) declares obesity as a risk factor for non-communicable diseases (NCD) [1,2], consisting of a multifactorial pathology and chronic low-grade inflammatory disease [3]. Obesity is a complex, heterogeneous and multifactorial morbid condition, to which both environmental and genetic factors contribute. It occurs due to an imbalance between caloric intake and energy expenditure, with consequent accumulation of an excess of adipose tissue, such as to increase the risk of morbidity and mortality of the individual himself [4]. As adiposopathy consisting not so much in weight gain rather in excess fat mass [5], the Body Mass Index (BMI) cannot define obesity [6,7], and it is not sufficient to evaluate the risk of developing a chronic disease. Therefore, obesity must be evaluated with tools that measure the amount of fat mass and consider the relationship between environment-genes and metabolic diseases [8].

Obesity may worsen metabolic disease in adverse endocrine and immune responses triggered by body fat dysfunction [9]. Among the most known pathologies caused by fat mass accumulation, there is dyslipidemia and cardiovascular diseases (CVDs) [10].

According to the most recent guidelines on the management of obesity, prevention must start from therapeutic lifestyle changes, which include the prescription of personalized diet therapy following the evaluation of the nutritional status, the prescription of a physical activity (PA) program and behavioural therapy to maintain and strengthen patient’s adherence to the treatment [11].

A key goal of cardiovascular disease prevention efforts is to predict events over medium to long periods of time [12]. Valid predictive indexes of cardiovascular and Myocardial Infarction (MI) risk, atherosclerosis and coronary heart disease have been determined: the total cholesterol/high-density lipoprotein cholesterol (cHDL) ratio [13], the triglycerides (TG)/cHDL ratio [14]; the low-density lipoprotein cholesterol (cLDL)/cHDL ratio [15]; the Atherogenic Index of Plasma (AIP) [16]; the Lipid Accumulation Product (LAP) [17,18]; Fatty Liver Index (FLI) [19]. Among different inflammatory markers, such as C-Reactive Protein (CRP) and Erythrocyte Sedimentation Rate (ESR) [20], the evaluation of Neutrophil/Lymphocyte Ratio (NLR) and the Platelet/Lymphocyte Ratio (PLR) may have a high value in predicting the prognosis of different CVDs including MI, acute coronary syndrome, Heart Failure (HF) and Atherosclerosis [21,22]. Moreover, to predict the risk and recommend management strategies for those at risk of Atherosclerotic Cardiovascular Diseases (ASCVD), a specific Risk Algorithm was standardized (ASCVD risk Algorithm) [23,24]. In addition, the BARD score (BMI, Alanine aminotransferase (ALT)/Aspartate aminotransferase (AST) ratio (AAR), Diabetes Mellitus (DM)), one of the most used algorithms for fibrosis evaluation, could be useful to predict a CVDs risk linked to the cHDL value in patients with hepatic fibrosis [25,26]. 

The guidelines of the American College of Cardiology/American Heart Association (ACC/AHA) for the prevention of ASCVD focus on the evaluation of cardiovascular risk [23,24], the implementation of a healthy lifestyle, the management of overweight and obesity and the treatment of high blood pressure and hypercholesterolemia.

Di Renzo et al. highlighted the personalization of the Mediterranean Diet (MD) plays a key role in the prevention and treatment of Non-Communicable Chronic Diseases (NCCDs) [11]. Moreover, numerous studies evaluated the role of MD on different parameters and confirmed that adherence to the MD was associated with a reduced incidence, prevalence and mortality from coronary heart disease, as well as with other CVDs and a reduced all-cause mortality [27]. As reported by Martini et al., the positive action of MD on the cardiometabolic risk was due to the capability of decreasing the risk of diabetes and metabolic-related conditions [28]. Study on gene–diet interaction showed that MD represented a good nutritional treatment to reduce the body fat mass [29]. Moreover, several parameters, i.e., BMI, waist circumference, waist-to-hip (WHR) ratio, liver enzymes, serum glucose and CVDs risk indexes, were impaired by MD and a more active lifestyle [30].

For a therapeutic change in lifestyle, in addition to a healthy diet, an increase in motor activity is also necessary, from walking to endurance activity. Regular PA is indicated to either prevent or delay the onset of CVDs [31]. Different types of PA, increased walking, and to a lesser extent, exercise intensity, were independently associated with a lower risk of CVDs.

Considering this scientific evidence, the main aim of this prospective observational study was to evaluate, for the first time, the effectiveness of a combined MD therapy intervention and a PA plan, on the adipocyte risk indexes AIP, LAP, BARD score and FLI, in a population of women at risk of CVDs and with different pathological conditions.

The secondary purpose was to evaluate the effects on cardiovascular and inflammatory risk indexes (ASCVD, total cholesterol/cHDL, TG/cHDL, cLDL/cHDL, NLR and PLR), the changes of the body composition, evaluated through anthropometry, Dual-energy X-ray absorptiometry (DXA) and Bioelectrical Impedance Analysis (BIA).

## 2. Materials and Methods

### 2.1. Subjects and Study Design

The prospective observational study in a single cohort of adult women, at the Section of Clinical Nutrition and Nutrigenomics, Department of Biomedicine and Prevention of the University of Rome Tor Vergata (Rome, Italy) was conducted between May 2018 and July 2019. We consecutively enrolled all the women, who voluntarily came up at the Section of Clinical Nutrition and Nutrigenomics, Department of Biomedicine and Prevention of the University of Rome Tor Vergata, for nutritional-medical check-up.

To be eligible, each woman had to be Caucasian, Italian, older than 18 years old and had to be affected by at least one pathological condition in absence of drug treatment (obesity, pre-diabetes, diabetes, metabolic syndrome, osteopenia, arterial hypertension, dyslipidemia, ischemic heart disease, hepatosteatosis, hyperuricemia, Obstructive Sleep Apnea Syndrome (OSAS), chronic kidney disease). Patients with a diagnosis of cancer, hepatitis, viral infections, underweight or in therapy with antioxidant supplements were excluded.

All the patients had a specialist nutritional consultation for the evaluation of their nutritional status at baseline (T0), and after 6 months (T1). The nutritional evaluation consisted of a medical examination, anthropometric measurements, laboratory tests, the determination of body composition analysis carried out using both BIA (BIA 101S, Akern/RJL Systems, Pontassieve, Florence, Italy) and DXA (I-DXA, GE Medical Systems, Milwaukee, WI, USA). All the measurements were performed after a 12 h overnight fast.

For what concerns weight, the population was divided according to a loss of the initial body weight equal to or greater than 10% and lower than 10%. The choice of the cut-off of 10% was taken in relation to the well-known data of the scientific literature according to which a 10% reduction of the initial body weight is sufficient to reduce the risk of complications and mortality from NCCDs [32]. Conversely, concerning the FM loss from the DXA analysis, the cut-off of 15% was chosen considering the sample’s median fat loss at T1.

A personalized dietetic plan and a scheduled physical activity were prescribed for each patient and the follow-up consultations were carried out after 6 months. To ensure adherence to the diet and PA plan, patients were monitored during the 6 months by monthly telephone interviews or e-mails.

### 2.2. Body Composition Assessmen

#### 2.2.1. Anthropometric Assessment

After a 12-h overnight fast, all subjects underwent a body composition assessment. Anthropometric parameters were measured for all participants according to standard methods: body weight, height, hip and waist circumferences. Candidates were instructed to take off their clothes and shoes before performing all the measurements.

Body weight and height were evaluated using a scale and a stadiometer (Invernizzi, Rome, Italy), while the subject was standing and wearing underwear. Data were collected to the nearest 0.1 kg and 0.1 cm, respectively. BMI was calculated according to the following formula:

BMI = body weight/height^2^ (kg/m^2^)

Body circumferences (neck, abdomen, waist and hip) were measured with flexible and non-extensible metric tape [33], according to the International Society for the Advancement of Kinanthropometry protocol and National Institute of Health Guidelines [34].

The WHR was calculated as a predictor of MI risk [35].

#### 2.2.2. Bioelectrical Impedance Analysis (BIA)

BIA allowed for measuring the impedance of the human body to the passage of an alternating current, with constant intensity (400 μA) and frequency (50 KHz), according to the amount of water and electrolytes in the body. It generates Resistance (Rz) and Reactance (Xc) values for each patient that are processed through the BIA Akern software with validated algorithms, providing graphical and numerical data on the analysis of body composition [36].

BIA was used to evaluate resistance (Rz), reactance (Xc), hydration, total body water (TBW), extracellular water (ECW), intracellular water (ICW) and phase angle (PA).

#### 2.2.3. Dual-Energy X-ray Absorptiometry (DXA)

DXA was performed to assess total body FM (percentage and kg), total body lean mass (LM) (kg), and total body bone mass (TBBone) [7]. Before each session, the standard DXA instrument quality control and calibration measures were conducted. They laid supine on the DXA, without moving for 20 min when the DXA scan recorded their results. The coefficient of variation (CV% = 100 × SD/mean) intra and inter subjects ranged from 1% to 5%. The coefficient of variation for bone measurements was less than 1%; CVs on this instrument for five subjects scanned six times over a nine-month period were 2.2% for FM and 1.1% for lean body mass (LM). The radiation dose of the procedure was 0.01 mSv.

FM% was calculated as FM (kg) divided by the total mass of all tissues, including the LM and TBBone, as the following [37]:

FM% = (FM/(FM + LM + TBBone)) × 100.

In order to define muscle mass status the Appendicular Skeletal Muscle Mass Index (ASMMI) was calculated using the following formula [29,38]:

Legs Muscle Mass (kg) + Arms Muscle Mass (kg)/Height2 (m^2^).

Subjects with the percentage of total body fat mass (FM) < 30% were considered normal weight, otherwise they were considered pre-obese/obese [39].

### 2.3. Laboratory Tests, Cardiovascular and Inflammatory Risk Indexes

The evaluated parameters were blood count, glycemia, insulinemia, hepatic transaminases, γ-glutamyl transferase, creatinine, lipidemic profile (total cholesterol, cLDL, cHDL, TG), C-reactive protein (CRP) and Erythrocyte Sedimentation Rate (ESR).

Blood tests were prescribed by clinicians of the Section of Clinical Nutrition and Nutrigenomics, Department of Biomedicine and Prevention of the University of Rome Tor Vergata, and subjects were asked to perform them at the same accredited laboratory of Tor Vergata Hospital (Rome, Italy). To assess the cardiovascular and inflammatory risk, the following indexes and ratios were calculated.

Currently, the ACC/AHA guidelines, updated in June 2019, define severe hypercholesterolemia when the plasma Low-Density Lipoprotein cholesterol (cLDL) value is greater or equal to 190 mg/dL or 4.9 mmol/L. In this condition, the recommendation is to undergo high-intensity statin therapy immediately without calculating 10-year ASCVD risk [24].

To assess the cardiovascular and inflammatory risk, the following indexes and ratios were calculated:

(1) Total cholesterol/cHDL ratio was calculated according to the formula [13,40].
  Total cholesterol/cHDL ratio = Total cholesterol (mg/dL)/cHDL (mg/dL), (1)
  with normal values < 3.
(2) Lipoproteins cholesterol (cLDL/cHDL) ratio was calculated according to the formula [15]:
  cLDL/cHDL ratio = cLDL (mg/dL)/cHDL (mg/dL), (2)
  with normal values < 2.
(3) Triglycerides (TG) /cHDL ratio was calculated according to the formula [14]:
  TG/cHDL ratio = TG (mg/dL)/cHDL (mg/dL), (3)
  with normal values < 1.
(4) AIP was calculated according to the formula [16,41]:
  AIP = log (TG/cHDL)(4)
(5) The Fatty Liver Index (FLI) was calculated according to the formula [42]:
  FLI = (e0.953 × loge(triglycerides) + 0.139 × BMI + 0.718 × loge(ggt) + 0.053 × waistcircumference − 15.745)/(1 + e0.953 × loge(triglycerides) + 0.139 × BMI + 0.718 × loge(ggt) + 0.053 × waistcircumference − 15.745) × 100, (5)
  with absence of steatosis for values < 30.
(6) ASCVD Risk Algorithm was calculated using the ACC/AHA calculator [23].
(7) LAP was calculated according to the formula [17]:
  LAP = (WC-65) × TG for men and (WC-58) × TG for women.(6)
(8) BARD score consists of the weighted sum of three variables: body mass index ≥ 28 represents 1 point, the Aspartate Aminotransferase (AST)/Alanine Aminotransferase (ALT) ratio ≥ 0.8 represents 2 points, and diabetes mellitus represents 1 point. A score of 2–4 had an odds ratio of 17 (confidence interval: 9.2–31.9) to determine advanced fibrosis and a negative predictive value of 96% [43].
(9) NLR is easily calculated by dividing the absolute neutrophil count by the absolute lymphocyte count from a complete blood count with differential [22]. The values for Low risk < 1.6, Medium risk 1.6–2.4 and High risk > 2.4.
(10) PLR is calculated by dividing the platelet count by the lymphocytes [44]. The cut off is < 150.
(11) CRP and ESR were used to evaluate inflammatory risk [45].

### 2.4. Personalized Diet Therapy

At baseline, subjects were instructed to record weight and/or measures of foods and beverages consumed for a 3-day food intake assessment [46]. The estimated intake of macronutrients was calculated by using Dietosystem dietary software (DS Medica S.r.l., Milan, Italy).

Considering the results of the food intake, food tastes and calculating the daily energy expenditure, a personalized MD was prescribed to each patient. The diet was personalized, considering a caloric restriction of 20% compared to the daily energy expenditure for overweight and obese patients, while an isocaloric diet was prescribed to normal weight ones. The daily energy expenditure was calculated estimating Basal Metabolism according to the De Lorenzo Formula [47].

Daily macronutrient intake was distributed as follows: 55% of total kcal/day of carbohydrates, 20% of total kcal/day of protein (> 50% of vegetable derivation), < 25% of total kcal/day of lipids (on total daily energy intake: saturated fat < 10%, 6–10% polyunsaturated fatty acids (PUFA), *n*-6/*n*-3 PUFA ratio of 3:1, 15% of monounsaturated fatty acids (MUFA); < 1% trans-fatty acids) and 25 g of fiber.

The MD was balanced starting from the daily protein amount, with a protein intake of 2 g/Kg of total LM, according to Colica et al. [48,49]. The diet therapy prescribed was formulated according to the MD model based on whole grains, fresh fruits and vegetables (5 portions per day), legumes, nuts and daily use of olive oil. Plant proteins and fresh fish were preferred to red meat. Processed foods were excluded from the diet. A small amount (125 g) of red wine was allowed once a day. The adherence to the diet therapy was assessed remotely through a complete dietetic anamnesis, including the 24 h recall method and a specific food and beverages consumption investigation.

### 2.5. Planned Physical Activity

The levels of physical activity (PA) in diverse domains, such as working activity, leisure time activity and sedentary activities and participation in organized sport, were evaluated for each patient, requesting them to answer the question of the Finnish Diabetes Risk Score (FINDRISC) [16]: “Do you exercise during your free time and/or for work for at least 30 min almost every day?” All patients were recommended to engage in physical activity for 150 min per week of moderate-intensity aerobic activity (50–70% Heart Rate max) and/or 90 min per week of high-intensity activity (>70% HR max) distributed in at least three days a week, with no more than 2 consecutive days between bouts of aerobic activity, according to the American College of Sports Medicine and the American Diabetes Association [50]. PA was considered moderate when the time/week spent was on 60 min. To estimate sedentary PA, we considered the h/day spent on sedentary behaviours. To estimate vigorous PA, we considered time/week spent on 20 min of high-intensity activity PA. The level of physical activity was monitored and self-reported by patients during the monthly interviews.

### 2.6. Statistical Analysis

Statistical analysis was performed using IBM SPSS Statistics V15.0 (SPSS, Chicago, IL, USA). The Kolmogorov–Smirnov test was performed to evaluate variables in distribution. Continuous variables are represented as mean and standard deviation. The paired t-test and the Wilcoxon signed-rank test were performed to compare normal and skewed continuous variables, respectively, between pre- (T0) and post- (T1) treatment. The McNemar test was performed to compare dichotomous data between T0 and T1. Pearson’s correlation analysis was carried out to evaluate a possible linear correlation between the risk indexes compared to the weight loss and the loss of body fat mass. Results were significant for *p*-value < 0.05.

### 2.7. Ethics Approval

All participants enrolled in the study approved their participation after studying and signing the informed consent, carried out in accordance with the Helsinki Declaration of 1975 as revised in 1983. The study protocol was approved by the ethical committee of the Calabria Region Center Area Section (Register Protocol No. 146 17/05/2018).

## 3. Results

### 3.1. Subjects

A total of 71 patients were invited to participate to the study. Of those, 11 declined the invitation, while 8 were not eligible because already in pharmacological treatment. Finally, 52 of them met the criteria for eligibility (age 47.3 ± 12.5 years) and completed the study after 6 months (T1). At baseline, the 94% of the studied population was obese, and comorbidities of obesity were highlighted: mainly prediabetes, metabolic syndrome, dyslipidemia and arterial hypertension. In total, 54% had MS, 31% pre-diabetes, 13% T2DM, 37% arterial hypertension, 69% dyslipidemia, 4% ischemic heart disease, 29% hepatosteatosis, 8% hyperuricemia, 15% OSAS, 73% hypovitaminosis D, 6% chronic kidney disease and 38% osteopenia.

Considering the subjects on the basis of the number of comorbidities, the prevalence of multimorbidity of the sample was also assessed on the basis of the number of pathologies found at baseline for each patient: 15% of the population had 2 comorbidities, 25% had 3, 10 % had 4, 17% had 5 and 6 and 4% had 7.

During the study, the patients showed a good compliance to the diet and PA prescription, according to the self-report data collected through the monthly interviews.

### 3.2. Anthropometry and Body Composition

Table 1 summarizes the anthropometric and body composition’s characteristics of the population both at T0 and T1. At T1, after the nutritional and physical activity intervention, weight, BMI, as well as all the circumferences, significantly decreased (*p* < 0.0001). The TBW was significantly reduced, in particular for what concerned the extracellular compartment.

Patients after diet and physical activity therapies achieved the best results in terms of body composition and laboratory parameters. Bone mineral density remained almost unchanged in this period and did not undergo statistically significant changes.

Moreover, the DXA analysis showed a statistically significant reduction of the total body FM (kg and %). Bone Mineral Density (BMD) has been maintained from T0 to T1 (data not shown). Finally, the loss of FM is associated with a slight decrease in LM (*p* < 0.0001), according to the reduction of ECW measured by BIA.

When dividing the population into two groups according to the percentage of weight loss (less than 10% or equal/greater than 10%) LM resulted to be significantly reduced in subjects with a weight loss ≥ 10%, corresponding to a mean value of 2.4 kg (*p* < 0.0001).

Conversely, when considering the population according to an FM loss greater than or equal to 15% or lower than 15% of the initial FM, we found LM to be significantly decreased in both groups at T1 (subjects ≥15% FM loss: −1.2 kg of LM, *p* < 0.005; subjects <15% of FM loss: −1.1 kg of LM, *p* < 0.0004).

### 3.3. Cardiovascular Risk Indexes

At T0 most of the study population showed altered risk indexes, especially the ones associated with plasma lipoproteins. In particular, 98%, 92% and 81% of them had an impaired TG/cHDL, Total cholesterol/cHDL and cLDL/cHDL ratio, respectively; while 46%, 35% and 32% of them showed altered AIP, PLR and ASCVD risk, respectively. At T1, all risk indexes showed a significant statistical reduction, except for NLR, PLR and BARD (Table 2).

**Figure 1:**
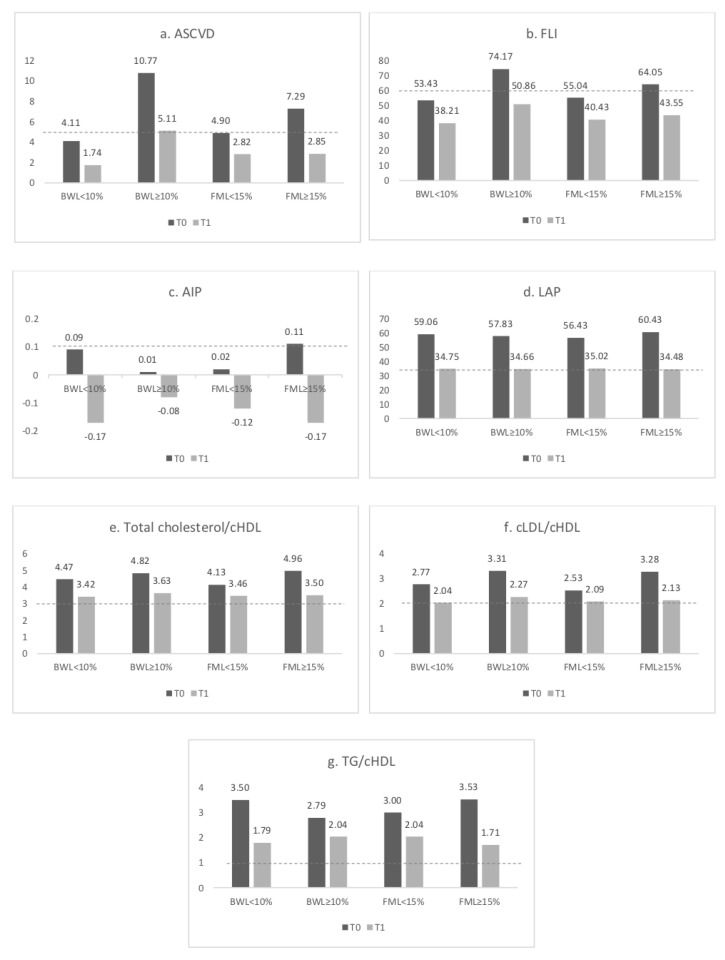
Modification of Cardiovascular Diseases and lipid profile-related risk indexes before (T0) and after the intervention (T1) according to Body Weight Loss (BWL) <10% and ≥10% and Fat Mass Loss (FML) <15% or ≥15%. (**a**) ASCVD, Atherosclerotic Cardiovascular Diseases; (**b**) FLI, Fatty Liver Index; (**c**) AIP, Atherogenic Index of Plasma; (**d**) LAP, Lipid Accumulation Product; (**e**) Total cholesterol/cHDL, total cholesterol-to-high density lipoprotein cholesterol ratio (**f**) cLDL/cHDL: low-density lipoprotein cholesterol-to-high density lipoprotein cholesterol ratio; (**g**) TG/cHDL, triglycerides-to-high density lipoprotein cholesterol ratio. The dotted lines refer to the cut-off for each index.

The ASCVD risk reduction was positively correlated to the decrease of both weight and FM (*p* < 0.0001, *p* = 0.004), regardless of the extent of the loss. When considering a reduction of body weight ≥ 10% and FM ≥ 15%, a switch from a higher to a lower risk class was observed (*p* < 0.0001 and *p* = 0.001, respectively). The FLI index significantly decreased with body weight and FM loss, regardless of the extent of the loss. Moreover, when considering a reduction of body weight ≥ 10% and FM ≥ 15 a switch from a higher to a lower risk class was observed (*p* < 0.0001). The AIP was significantly reduced with the loss of FM (*p* = 0.001), and more markedly with a loss greater than 15% (*p* < 0.0001). Conversely, a significant reduction of the AIP with a body weight loss of ≥ 10% (*p* = 0.1) was not observed. Finally, the LAP index significantly decreased with the reduction of body weight (*p* < 0.0001). The cardiovascular risk indexes associated with the lipid profile were significantly reduced with both the decrease of body weight and FM, regardless of the amount of decrease. A greater reduction was observed with a loss of FM ≥ 15% (*p* < 0.0001) (Figure 1).

## 4. Discussion

In recent literature, the CVDs risk reversibility has been widely demonstrated. Around 80% of cardiovascular events can be avoided by adopting a correct lifestyle consisting of eating healthy and staying physically active [51]. Nevertheless, the improvement of strategies aimed at controlling risk factors is still unsatisfactory today.

In the United States, in the context of modifiable risk factors, high blood pressure is the primary cause of atherosclerotic cardiovascular diseases [52].

Consequently, in all adults with high blood pressure, a non-pharmacological intervention is recommended in the first instance, while in hypertensive patients already in drug therapy, a pressure target of 130/80 mmHg must be pursued [53].

Therefore, it underlines the necessity of a multidisciplinary approach. It would be aimed at controlling risk factors through the implementation of preventive strategies, the importance of sharing decisions with patient and paying a deep look to the social determinants of health (obstacles to care, educational level, economic difficulties, environmental and socio-economic factors) [11].

The most recent epidemiological studies on nutrition have shown how dietary models instead of isolated nutrients can be a more accurate tool for studying eating habits and preparing a therapeutic plan for the prevention and treatment of NCD [54]. Healthy, personalized nutrition can reduce cardiovascular risk factors, such as obesity, diabetes, dyslipidemia and high blood pressure and therefore plays a crucial role in preventing the recurrence of chronic ischemic heart diseases [55]. Among dietary models, MD was associated with a lower risk of CVDs incidence and mortality, including coronary heart disease and MI [56].

Moreover, it has been shown that engaging in regular physical activity reduces the risk of developing and dying from cardiovascular outcomes [57,58].

However, to date, there are few clinical studies aimed at understanding the independent and even synergistic impacts of the effect of a dietary plan adhering to MD with physical inactivity [58].

Therefore, we set up a dietary and PA plan, aimed at improving the risk indexes of adiposity, inflammatory and cardio-vascular diseases, as well as weight loss of < 10% or ≥ 10% of the initial body weight and reduction of fat mass.

We analysed the statistical relationship between the improvement of risk indexes at baseline (T0) and after 6 months of therapy (T1). The reduction in the evaluated indexes was statistically associated with bodyweight regardless of the extent of the loss. The reduction of most of the evaluated indexes reflects a positive health change linked to the amelioration of laboratory tests from T0 to T1.

Considering the ASCVD risk index, which is a predictor for forecasting the potential impact of different interventions on patient risk [59], we observed an effective 10-year risk reduction for cardiovascular atherosclerotic disease when patients lost at least 10% of body weight or at least 15% of FM. In this context, the ASCVD risk reduction was strongly significant, leading to a transition from the higher to a lower risk category. Currently, the ACC/AHA guidelines, updated in June 2019, define severe hypercholesterolemia when the plasma cLDL value is greater or equal to 190 mg/dL or 4.9 mmol/L. In this condition, the recommendation is to undergo high-intensity statin therapy immediately without calculating 10-year ASCVD risk [14].

In our study, the dietary treatment with MD combined with a physical activity plan, resulted in a significant reduction of all blood parameters of the lipid profile, even in the absence of drug therapy.

In our study, we observed a statistically significant reduction of plasma lipoproteins, TG and total cholesterol, especially in subjects with reduced FM by at least 15%.

Total cholesterol/cHDL and cLDL/cHDL ratios are risk indicators with greater predictive value than isolated parameters used independently, particularly cLDL. The total cholesterol/cHDL ratio, known as the atherogenic or Castelli risk index [13], and the cLDL/cHDL ratio are two important components and indicators of cardiovascular risk, the predictive value of which is greater than the isolated parameters [11]. TG/cHDL ratio is a deputy marker of LDL particle size (small and dense) to observe the link with insulin resistance and thyroid co-morbidity [12].The reduction of most of the evaluated indexes reflects a positive health change linked to the amelioration of laboratory tests from T0 to T1.

The ratios between plasma lipoproteins showed a statistically significant reduction, thanks to the proper nutritional intervention, and were marked in subjects with reduced FM by at least 15%.

The same trend was also observed for the LAP, AIP and FLI index. Both LAP and FLI reflected the metabolic health of the patient. It is known that LAP is strongly correlated with visceral fat [60] and it is associated with metabolic syndrome [61], type-2 diabetes mellitus [17], hypertension [62] and CVDs [63]. FLI particularly is a simple and accurate predictor of hepatic steatosis in the general population [64]. Furthermore, AIP is a predictive indicator for the coronary artery disease in postmenopausal women [65] and it is a strong marker to predict the risk of atherosclerosis and coronary heart disease [40]. For what concerns the inflammation milieu, both NLR and PLR have the potential to be inflammatory markers that reflect the activity of many inflammatory diseases [66]. Obese subjects undergo a chronic inflammatory condition due to the adipose tissue dysfunction of immune-related activities, involving a transient infiltration of neutrophils within the abdominal fat and their binding to adipocytes. NLR and PLR are considered cost-effective markers for the detection of subclinical inflammation [67]. Nevertheless, the result was not statistically significant for the two indexes. This latter probably considering that dietary intervention should be followed for longer in order to show a higher effect on neutrophils, platelets, lymphocytes and liver transaminases.

The BARD score was established specifically for assessing and predicting Non-alcoholic Fatty Liver Disease (NAFLD). It is widely used to predict liver fibrosis in NAFLD patients and requires simple clinical data [68]. A BARD score of 2–4 was associated with an OR for advanced fibrosis of 17 (confidence interval 9.2 to 31.9) and a negative predictive value of 96% [26]. It was developed to exclude the presence of advanced fibrosis in patients with NAFLD [27]. However, in our study, the values of BARD score were in the moderate risk cut-off, and no significant changes were observed at T1 (*p* = 0.06).

After MD and PA therapy, we observed an improvement of the main anthropometric and body composition parameters. The LM remains stable in subjects who reduce FM <15%. In patients who lost more than 15% of FM, the reduction of LM observed could be linked to extracellular liquids, occurring mostly at the beginning of weight loss, and therefore also could be related to fat loss.

The proof of the above conditions can be demonstrated with the PA. Indeed, PA is a deep marker for sarcopenia, fragility and risk of mortality in obese people [69]. In our study, the PA reveals a slight improvement, even if it cannot be considered as significant data, due to the restricted period of intervention. Significant maintenance of LM after nutritional and physical interventions is undoubtedly a clinically important factor due to the preservation of muscle mass achieved to the dietetic nutritional composition in terms of proteins and amino acids. Future investigations should focus on ASMMI as a marker for the prevention of sarcopenia obesity [38]. It would be important to consider this effect when a nutritional and physical intervention is prescribed to a patient.

Finally, we can assume that the combination of diet therapy and PA lead to the best results in terms of body composition and laboratory tests.

The strength of the present study is that data are observed on a population with concomitant risk factors, and that it is the first one monitoring AIP, LAP and FLI during a combination between MD personalized diet therapy and PA program. Conversely, the main limitations are the small sample size, the lack of a longer follow-up and the self-report data about diet and PA adherence.

## 5. Conclusions

In conclusion, our findings suggest that a combination of both personalized MD diet therapy and PA program lead to body weight loss, body composition remodelling and risk indexes reduction. The evaluation of specific pathologies risk indexes, such as adiposity (AIP, LAP, FLI) and CVDs risk indexes, may represent a predictor factor to guide clinicians in the nutritional status assessment and improve the personalization of diet and PA prescription. As the main limitations of the study are the small sample size and the lack of a longer follow-up, additional investigations are needed in this field. Furthermore, future clinical studies should apply innovative and objective physical activity measurement methods, during the time of examination.

## Figures and Tables

**Table 1:** Anthropometric and body composition’s characteristics at T0 and T1.

	T0	T1	*p*-Value *
Weight	88.4 ± 24.9	79.7 ± 18.7	<0.0001
BMI	32.3 ± 8.0	29.2 ± 6.0	<0.0001
Neck circumference	38.8 ± 4.4	37.1 ± 3.9	<0.0001
Waist circumference	98.6 ± 18.0	90.7 ± 13.6	<0.0001
Abdomen circumference	109.3 ± 19.6	100.0 ± 13.2	<0.0001
Hip circumference	113.3 ± 14.6	107.3 ± 11.1	<0.0001
WHR	0.869 ± 0.098	0.846 ± 0.093	0.0002
Rz	470 ± 79	468 ± 76	0.85
Xc	50 ± 10	52 ± 10	0.21
PA°	6.17 ± 1.11	6.38 ± 1.07	0.1
TBW (L)	42.6 ± 9.3	41.5 ± 8.6	0.004
TBW (%)	49.3 ± 7.3	52.4 ± 6.7	<0.0001
ECW (L)	19.2 ± 4.1	18.3 ± 3.7	0.003
ECW (%)	45.4 ± 5.0	44.5 ± 4.6	0.11
ICW (L)	23.4 ± 6.1	23.3 ± 5.8	0.8
ICW (%)	54.6 ± 5.0	55.5 ± 4.6	0.10
FM (kg)	36.75 ± 17.16	29.80 ± 12.24	<0.0001
FM (%)	40.3 ± 9.1	36.5 ± 8.8	<0.0001
LM (kg)	48.56 ± 10.71	47.42 ± 10.14	<0.0001
ASMMI	8.32 ± 1.82	8.06 ± 1.71	0.003

ASMMI, appendicular skeletal mass index; BMI, body mass index; ECW, Extra-Cellular Water; FM, fat mass; ICW, intracellular index; LM, lean mass; PA, Phase Angle; Rz, Resistance; TBW, Total Body Water; WHR, waist-to-hip ratio; Xc, Reactance. * The paired *t*-test and the Wilcoxon signed-rank test were performed in order to compare normal and skewed continuous variables, respectively, between pre- (T0) and post- (T1) treatment. Statistical significance for *p* < 0.05.

**Table 2:** Modification of risk markers after nutritional intervention and prescription of physical activity.

Cardiovascular Risk Indexes	T0	T1	*p* Value *
ASCVD risk	6.27 ± 7.21	2.84 ± 3.44	0.0001
NLR	1.73 ± 0.74	1.84 ± 0.57	0.31
PLR	116.24 ± 37.58	125.78 ± 65.29	0.29
Total cholesterol/cHDL	4.64 ± 1.38	3.50 ± 0.87	<0.0001
cLDL/cHDL	2.95 ± 1.09	2.12 ± 0.69	<0.0001
TG/cHDL	3.38 ± 2.53	1.89 ± 1.07	<0.0001
AIP	0.06 ± 0.27	−0.14 ± 0.22	<0.0001
FLI	59.74 ± 32.26	42.06 ± 30.73	<0.0001
LAP	58.66 ± 42.79	34.72 ± 23.43	<0.0001
BARD	3.42 ± 0.11	2.89 ± 0.23	0.06

ASCVD, Atherosclerotic Cardiovascular Diseases, AIP, Atherogenic Index of Plasma; BARD, (BMI, Alanine aminotransferase (ALT)/Aspartate aminotransferase (AST) ratio (AAR), Diabetes Mellitus (DM), cLDL/cHDL, low-density lipoprotein cholesterol-to-high density lipoprotein cholesterol ratio; FLI, Fatty Liver Index; LAP, Lipid Accumulation Product; NLR, neutrophils-to-lymphocytes ratio; PLR, platelets-to-lymphocytes ratio; Total cholesterol/cHDL, total cholesterol-to-high density lipoprotein cholesterol ratio; TG/cHDL, triglycerides-to-high density lipoprotein cholesterol ratio. * The paired t-test was performed to compare continuous variables between pre- (T0) and post- (T1) treatment. Statistical significance for *p* < 0.05.
